# SUN11602, a bFGF mimetic, modulated neuroinflammation, apoptosis and calcium-binding proteins in an in vivo model of MPTP-induced nigrostriatal degeneration

**DOI:** 10.1186/s12974-022-02457-3

**Published:** 2022-05-07

**Authors:** Alessio Ardizzone, Valentina Bova, Giovanna Casili, Alessia Filippone, Michela Campolo, Marika Lanza, Emanuela Esposito, Irene Paterniti

**Affiliations:** Department of Chemical, Biological, Pharmaceutical and Environmental Sciences, University of Messina, Viale Ferdinando Stagno D’Alcontres, 31, 98166 Messina, Italy

**Keywords:** Parkinson’s disease (PD), SUN11602, MPTP-mouse model, Neuroprotective, Calbindin, Ca^2+^ homeostasis

## Abstract

**Background:**

Parkinson’s disease (PD) is the second most frequent neurodegenerative disease. PD etiopathogenesis is multifactorial and not yet fully known, however, the scientific world advised the establishment of neuroinflammation among the possible risk factors. In this field, basic fibroblast growth factor/fibroblast growth factor receptor-1 (bFGF/FGFR1) could be a promising way to treat CNS-mediated inflammation; unfortunately, the use of bFGF as therapeutic agent is limited by its side effects. The novel synthetic compound SUN11602 exhibited neuroprotective activities like bFGF. With this perspective, this study aimed to evaluate the effect of SUN11602 administration in a murine model of MPTP-induced dopaminergic degeneration.

**Methods:**

Specifically, nigrostriatal degeneration was induced by intraperitoneal injection of MPTP (80 mg/kg). SUN11602 (1 mg/kg, 2.5 mg/kg, and 5 mg/kg) was administered daily by oral gavage starting from 24 h after the first administration of MPTP. Mice were killed 7 days after MPTP induction.

**Results:**

The results obtained showed that SUN11602 administration significantly reduced the alteration of PD hallmarks, attenuating the neuroinflammatory state via modulation of glial activation, NF-κB pathway, and cytokine overexpression. Furthermore, we demonstrated that SUN11602 treatment rebalanced Ca^2+^ overload in neurons by regulating Ca^2+^-binding proteins while inhibiting the apoptotic cascade.

**Conclusion:**

Therefore, in the light of these findings, SUN11602 could be considered a valuable pharmacological strategy for PD.

**Supplementary Information:**

The online version contains supplementary material available at 10.1186/s12974-022-02457-3.

## Background

Neurodegenerative diseases represent a broad clinical picture of central nervous system (CNS) disorders, in which inflammation and neuronal cell death are key features [1, 2]. In this field, Parkinson’s disease (PD) is the second most common neurodegenerative disease [3], characterized by motor and non-motor symptoms that lead to highly debilitating living conditions for patients [4]. Every year 5 million people are affected by PD [5], however, despite this wide diffusion its etiopathogenesis is not yet fully known.

Several PD pathological hallmarks were identified, in particular, PD patients showed a marked loss of dopaminergic neurons in the substantia nigra pars compacta and an extensive accumulation of misfolded α-synuclein, a major component of intracytoplasmic inclusions called Lewy bodies [6].

Moreover, in the last decade, growing evidences [7, 8] validates the involvement of neuroinflammation in PD pathophysiology, thus revealing the mechanisms underlying neuronal damage and also providing new insights into disease management.

In this regard, numerous studies [9, 10] highlighted the neuroprotective activity of basic fibroblast growth factor (bFGF; FGF-2), an endogenous ligand of the membrane receptor fibroblast growth factor type 1 (FGFR1) [11]. The neuroprotective effect of the bFGF/FGFR1 axis in counteracting neuroinflammation is closely related to the Ca2^+^ intracellular metabolism and Ca2^+^ binding proteins, such as Calbindin-D28k [12, 13].

Nevertheless, the therapeutic use of bFGF is limited by its short half-life in the blood and poor blood–brain barrier permeability [14] as well as by its side effects, such as a reduction in blood pressure and an increase in proliferative activity during treatment [15, 16]. To overcome these limitations SUN11602, an aniline-derived compound, was synthetized.

As reported by Ogino et al. [14] and Murayama et al. [16] this molecule exhibited excellent pharmacokinetic properties, in fact, after oral administration the bioavailability was greater than 65% in all the animals analyzed, like rats, mice and dogs (unpublished data).

SUN11602 interacts with the FGFR1 receptor similarly to bFGF, thus enhancing its phosphorylation, stimulating neuronal growth and survival in pathological conditions.

Furthermore, the FGFR1 activation induced by SUN11602 increases the expression of Calbindin-D28k, a Ca2^+^ binding protein widely expressed in many areas of the brain, and involved in the maintenance of Ca2^+^ intracellular homeostasis [16].

Therefore, this study aimed to evaluate the neuroprotective role of SUN11602 in counteracting neuroinflammatory processes and Parkinson's markers in a mouse model of MPTP-induced dopaminergic neurodegeneration.

## Materials and methods

### Materials

SUN11602 was purchased by Tocris Bioscience (Bristol, UK); detailed datasheets are available from the manufacturer’s website. Unless otherwise indicated all compounds were obtained from Sigma-Aldrich Company Ltd. (Milan, Italy). All other chemicals were of the highest commercial grade available. All stock solutions were prepared in non-pyrogenic saline (0.9% NaCl; Baxter, Italy, UK).

### Animals

Adult male CD1 mice (30–35 g; 6–8 weeks old; Envigo, Italy) were accommodated in a controlled environment and provided with standard rodent chow and water. Mice were housed in stainless steel cages in a room maintained at 22 °C ± 1 °C with a 12 h light and 12 h dark cycle. Animal experiments are in compliance with Italian regulations on the protection of animals used for experimental and other scientific purposes (DM 116,192) as well as EU regulations (OJ of EC L 358/1 12/18/1986) and the ARRIVE guidelines. Moreover, the study was approved by the OPBA of Messina with authorization number 537/2018-PR.

### MPTP-induced nigrostriatal degeneration

Adult male CD1 mice received four intraperitoneal injections of MPTP (20 mg/kg; Sigma-Aldrich, St. Louis, MO) in saline at 2 h intervals in 1 day, the total dose for each mouse was 80 mg/kg. Starting 24 h after the first MPTP injection, animals received oral administration of SUN11602 at doses of 1–2.5–5 mg/kg (in 10% DMSO), respectively; thereafter, oral administration was given once daily until 7 days after the MPTP injection. Mice were killed 7 days after MPTP injection and their brains were harvested, sectioned, and processed. The dose of MPTP (80 mg/kg) used was based on previous in vivo studies [17, 18].

### Experimental groups

Animals were randomly distributed into the following groups:

- Group 1: Sham + vehicle; vehicle solution (saline) was injected intraperitoneally during the first day like MPTP group, in addition, starting 24 h after vehicle injection, saline was administered orally for 7 consecutive days (*N* = 10);
- Group 2: Sham + SUN11602 1 mg/kg; like Sham + vehicle group, in addition mice was administered orally with SUN11602 1 mg/kg for 7 consecutive days starting 24 h after vehicle solution injection (*N* = 10);
- Group 3: Sham + SUN11602 2.5 mg/kg; like Sham + vehicle group, in addition mice was administered orally with SUN11602 2.5 mg/kg for 7 consecutive days starting 24 h after vehicle solution injection (*N* = 10);
- Group 4: Sham + SUN11602 5 mg/kg; like Sham + vehicle group, in addition mice was administered orally with SUN11602 5 mg/kg for 7 consecutive days starting 24 h after vehicle solution injection (*N* = 10);
- Group 5: MPTP + vehicle: MPTP solution was administered intraperitoneally during the first day, as described before, plus saline oral administration for 7 consecutive days starting 24 h after MPTP injection (*N* = 10);
- Group 6: MPTP + SUN11602 1 mg/kg; like MPTP + vehicle group, in addition mice was administered orally with SUN11602 1 mg/kg for 7 consecutive days starting 24 h after MPTP injection (*N* = 10);
- Group 7: MPTP + SUN11602 2.5 mg/kg; like MPTP + vehicle group, in addition mice was administered orally with SUN11602 2.5 mg/kg for 7 consecutive days starting 24 h after MPTP injection (*N* = 10);
- Group 8: MPTP + SUN11602 5 mg/kg; like MPTP + vehicle group, in addition mice was administered orally with SUN11602 5 mg/kg for 7 consecutive days starting 24 h after MPTP injection (*N* = 10).

The dose and route of administration of SUN11602 were based on previous in vivo studies [14, 17], on a large-scale dose studies performed in our laboratory, and considering the mice body surface area-based dosing.

The experimental data relating to the Sham groups treated with SUN11602 were reported but showed only in the preliminary results (behavioral test), as they did not determine either toxicity or improvement compared to the Sham + vehicle group.

### Behavioral testing

Behavioral assessments on each mouse were made 7 days after the last MPTP injection.

The mice were placed in the behavior room for 5 min for 2 days for acclimation prior to the onset of behavioral testing [17].

#### Pole test

The pole test was performed to assess movement disorders. The test consisted of a 50 cm high, gauzed pole (1 cm in diameter). The animals were placed on top of a vertical pole, directed towards their cages. Under natural conditions, the mice will be oriented downwards along the length of the pole. The parameters evaluated in this test were: time until the animal turned by 180°, called the turning time, and time until the animal dropped to the floor, total time [17].

#### Elevated plus maze (EPM)

Anxiety deficits were evaluated using elevated plus maze (EPM) system. We used the same methodology employed in our previous study [17] and briefly described below. The elevated plus-maze test is one of the most used tests to measure anxiety-related behavior in rodent animals. The apparatus consists of two open arms, two closed arms and a center area. Mice were placed individually in the open arm, the time allowed to explore was 5 min. Behavioral representative of animal’s emotional state were: latency, frequency and duration of visits in the open and closed arms. The total number of entries in the arms was used as a general index of activity. Data were analyzed by One-way analysis of variance, with the percentage of time spent in the closed arms as a percentage of the total.

### Immunohistochemical localization of TH, DAT, α-syn and MAP-2

Immunohistochemical staining was performed as previously described [19, 20] and reported below. The brains were removed, fixed and embedded in paraffin. The brain sections of 7 µm were used for immunohistochemical staining. Briefly, after deparaffinization, endogenous peroxidase was quenched with 0.3% hydrogen peroxide in 60% methanol for 30 min. Sections were incubated overnight (O/N) with following antibodies: anti-Tyrosine hydroxylase (TH) (1:100; sc25269; Santa Cruz Biotechnology), anti-dopamine transporter (DAT) (1:100; sc-14002; Santa Cruz Biotechnology), pan-anti α-synuclein (α-syn; affinity purified rabbit polyclonal antibody raised against a peptide mapping at the C-terminus of α-synuclein of human origin) (1:100; sc-7011; Santa Cruz Biotechnology) and anti-MAP-2 (1:100; SB5622; Millipore). Subsequently, the sections were washed with PBS and incubated with secondary antibody for 1 h. The signal of binding with the antibodies was amplified by using the peroxidase Avidin–Biotin complex (Vector Lab. Inc., Burlingame, CA). The reaction was revealed by a chromogenic substrate (brown DAB), and counterstaining with Nuclear Fast Red. To prove the binding specificity for different antibodies, some sections were also incubated with only primary antibody or secondary antibody, no positive staining was observed in these sections. Images were collected using a Zeiss microscope and Axio Vision software. For graphic display of densitometric analyses, the % of positive staining (brown staining) was measured by computer-assisted color image analysis (Leica QWin V3, UK). All stained sections were observed and analyzed in a blinded manner. For immunohistochemistry, 20 × (50 µm scale bar) and 40 × (20 µm scale bar) were shown.

### Immunofluorescence staining of GFAP, IBA1, β3-tubulin and p-53

Brain sections were processed for immunofluorescence staining as previously described [21] and reported below. Sections were incubated with primary anti-GFAP antibody (1:100; sc-33673; Santa Cruz Biotechnology), anti-IBA1 antibody (1:100; sc-32725; Santa Cruz Biotechnology), anti-β3-tubulin antibody (1:100; sc-69966; Santa Cruz Biotechnology) and anti-p53 antibody (1:100; sc-126; Santa Cruz Biotechnology) in a humidified chamber for O/N at 37 °C. After washing with PBS solution, sections were incubated with IgG (H + L) Highly cross-adsorbed goat anti-mouse secondary antibody, Alexa Fluor™ (1:1000 in PBS v/v, Molecular Probes, Altrincham, UK), Invitrogen, for 1 h at 37 °C. Subsequently, brain sections were washed in PBS and nuclear staining with 4′,6′-diamidino-2-phenylindole (DAPI; Hoechst, Frankfurt, Germany) (2 µg/mL) in PBS was added. Sections were observed and acquired at 40 × magnifications using a Leica DM2000 microscope (Leica, UK, EU). All images were digitalized at a resolution of 8 bits into an array of 2560 × 1920 pixels. Optical sections of fluorescence specimens were obtained using a HeNe laser (543 nm), a laser UV (361–365 nm) and an argon laser (458 nm) at a 1 min, 2 s scanning speed with up to 8 averages; 1.5 μm sections were obtained using a pinhole of 250. Contrast and brightness were established by examining the most brightly labeled pixels and applying settings that allowed clear visualization of structural details while keeping the highest pixel intensities close to 200. The same settings were used for all images obtained from the other samples that had been processed in parallel. Digital images were cropped and figure montages prepared using Adobe Photoshop CS5 (Adobe Systems; Palo Alto, CA, USA). Cell counting analysis was made on ventral mesencephalon brain slices for a total of three slices per animal (*n* = 10 for each group). All stained sections were observed and analyzed in a blinded manner.

### Western blot analysis

During dissection, brains were surgically removed, and the ventral mesencephalon was isolated as previously described by Campolo et al. [17].

Tissue samples were processed as previously reported [22]. Cytosolic and nuclear extracts were obtained by treating the brain section with Buffer A and Buffer B containing protease inhibitors, respectively. The expression of IĸB-α, TNF-α, IL-1β, IL-6, IL-18, Calbindin-D28k, S100-β, Bcl-2, Bax and Caspase-3 were quantified in cytosolic fractions. NF-ĸBp65 was quantified in the nuclear fraction. The membranes were incubated, at 4 °C overnight, with specific primary antibodies: anti-IĸB-α (1:500; sc-1643; Santa Cruz Biotechnology), anti-TNF-α (1:500; sc-52746; Santa Cruz Biotechnology), anti-IL-1β (1:500; sc-32294; Santa Cruz Biotechnology), anti-IL-6 (1:500; sc-130326; Santa Cruz Biotechnology), anti-IL-18 (1:500; sc-7954; Santa Cruz Biotechnology), anti-Bcl-2 (1:500; sc-7382; Santa Cruz Biotechnology), anti-Bax (1:500; sc-7480; Santa Cruz Biotechnology), anti-Caspase-3 (1:500; sc-7272; Santa Cruz Biotechnology), anti-Calbindin-D28k (1:500; sc-28285; Santa Cruz Biotechnology), anti-S100-β (sc-393919; Santa Cruz Biotechnology) and NF-κB (1:500; sc-8008; Santa Cruz Biotechnology) in 1 × phosphate-buffered saline (PBS), 5% (w/v), non-fat dried milk and 0.1% Tween-20. Thereafter, the membranes were washed and incubated with secondary antibody (1:1000, Jackson ImmunoResearch, West Grove, PA, USA) for 1 h at room temperature. To confirm that the samples used contained a uniform protein concentration, membranes were incubated with primary anti-β-actin antibody (1:500; sc-47778; Santa Cruz Biotechnology, Dallas, TX, USA) for the cytosolic fraction or LAMIN A/C (1:500; sc-376248; Santa Cruz Biotechnology, Dallas, TX, USA) for the nuclear fraction.

Signals were detected by enhanced chemiluminescence (ECL) detection system reagent according to the manufacturer’s instructions (SuperSignal West Pico Chemiluminescent Substrate, Thermo Fisher Scientific, Waltham, MA, USA). The relative expression of the protein bands was quantified by densitometry with BIORAD ChemiDoc™XRS + software and standardized to β-actine or LAMIN A/C levels as internal control.

### Measurement of dopamine, 3,4-dihydroxyphenylacetic acid (DOPAC), and homovanilic acid (HVA) levels in the striatum

Measurements were made as previously showed [18, 23] and briefly reported below. 7 days after the last MPTP injection, 4 mice per group, were killed and the striatum was dissected, frozen on dry ice and stored at − 70 °C. To measure the levels of Dopamine and its metabolites, DOPAC and HVA, high performance liquid chromatography (HPLC) with electrochemical detection was used in each sample, using 0.15 M monochloroacetic acid, pH 3.0 and 200 mg/L Sodium octyl sulfate, 0.1 mM EDTA, 4% acetonitrile and 2.5% tetrahydrofuran as mobile phase. Data were collected and processed on a Dynamax computerized data manager (Rainin Instruments).

### Calpain activity

Calpain activity was estimated fluorometrically according to previous studies [24, 25]. Briefly, the supernatant of the brain samples, containing calpain, was subjected to Ca-dependent fluorescence and non-Ca-dependent fluorescence, to determine calpain activity, using N-succinyl-Leu-Tyr-(N-succinyl-LY)-AMC, cleaved by µ/m-calpain. To measure Ca-dependent fluorescence, samples were incubated at 37 °C in buffer A containing 63 mm imidazole–HCl, pH 7.3, 10 mm B-mercaptoethanol and 5 mm CaCl2 and cleaved with 150um M-succinyl-LY-AMC. The same methodology was performed to measure non-Ca-dependent fluorescence, using Buffer A without calcium, containing 1 mm EDTA and 10 mm EGTA.

### Stereological analysis

Unbiased counting of TH^+^ dopaminergic neurons within substantia nigra par compacta (SNpc) was performed as described previously [26, 27]. Each section was incubated with polyclonal primary antibody mouse anti-TH (1:400, Santa Cruz Biotechnology) O/N and processed with the ABC method (Vector Laboratories, Burlingame, CA). Brain sections were counterstained with cresyl violet, a Nissl stain, and covered. To count the number of TH^+^ cells, StereoInvestigator software was used (Microbrightfield, Williston, VT). Cells were counted with a 10 × and 20 × objective, respectively, using a Leica DM2000 microscope (Leica, UK, EU). The area of interest for counting TH-immunoreactive cells was performed within a 50 × 50 × 5 µm frame on the same side of the brain, with an upper and lower control zone of 1 µm; for Nissl cell counting, the same sections were examined.

### ELISA kit

Phospho-α-syn (p-α-syn) and CD68 were evaluated on brain tissues extracts for each experimental group as previously described [20].

phospho-α-syn (p-α-syn) and CD68 were measured by ELISA kit, according to the manufacturer's instructions (MyBioSource, respectively), through a colorimetric microplate reader.

### Statistical analysis

Experimental data are expressed as mean ± standard error of the mean (SEM) of N observations, in which *N* represents the number of animals studied. Data are representative of at least three independent experiments. The results were examined by one-way ANOVA analysis of variance followed by a Bonferroni post hoc test for multiple comparisons. Only a *p*-value less than 0.05 was considered significant.

## Results

### SUN11602 administration ameliorated behavioral impairments induced by MPTP intoxication

**Fig. 1:**
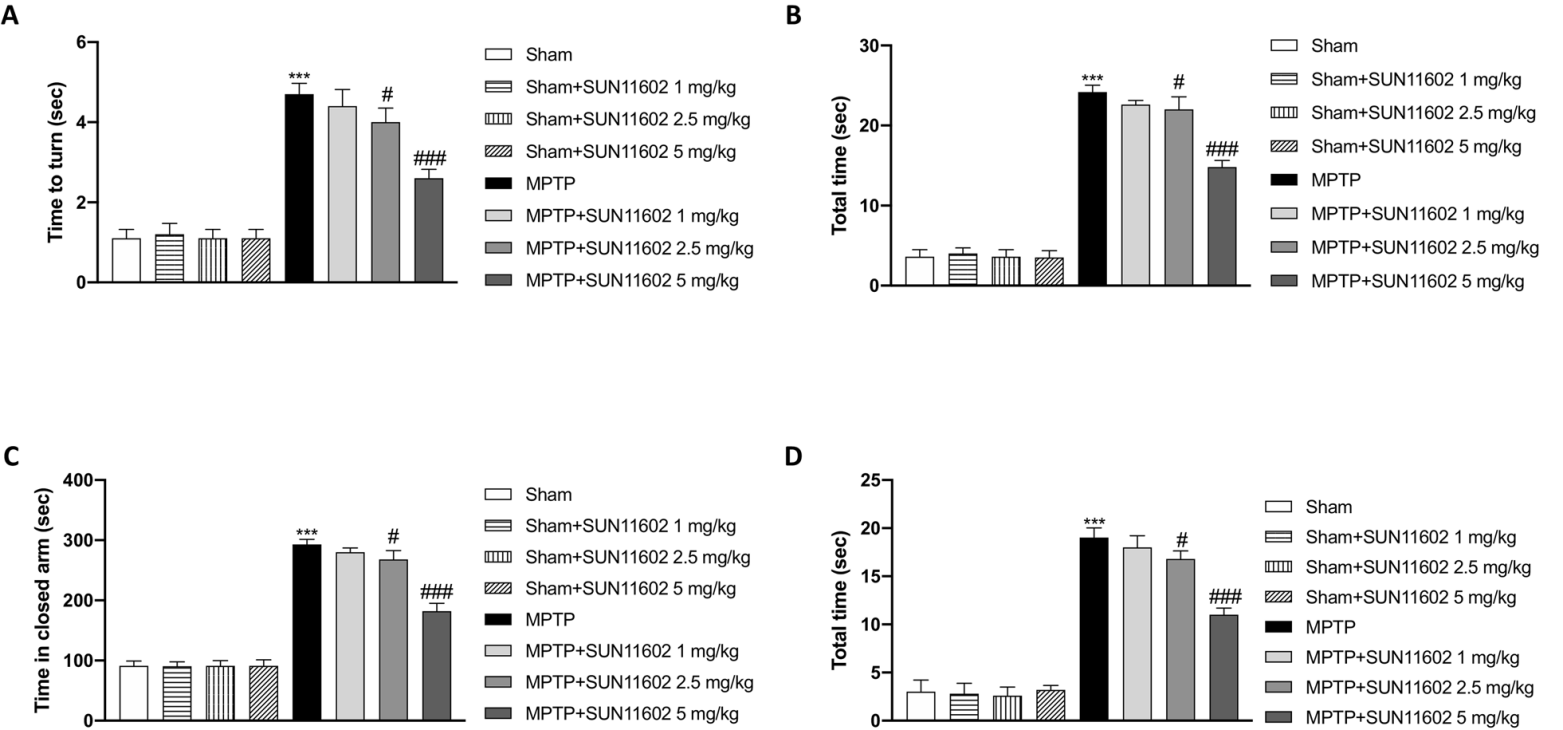
Effect of SUN11602 on behavioral impairments induced by MPTP. 7 days after MPTP injection mice showed a significant increase in behavioral deficits compared to the Sham group (**A**–**D**). Contrarily, SUN11602, at the two highest doses, considerably decreases “Time to turn” and “Time in closed arms” (**A**–**D**). Sham + SUN11602 administered groups were comparable to Sham + vehicle animals (**A**–**D**). Data are representative of at least three independent experiments. Values are means ± SEM. One-way ANOVA test. ***p < 0.001 vs Sham; ^#^*p* < 0.05 vs MPTP; ^###^*p* < 0.001 vs MPTP

Behavioral tests were performed in all the experimental groups. The pole test was performed to evaluate the motor alteration and bradykinesia caused by MPTP intoxication. The pole test showed that “Time to turn” and “Total time” increased in MPTP-injected mice compared to the Sham group (Fig. 1A and B). SUN11602 5 mg/kg administration showed an important decrease of “Time to turn” and “Total time” compared to the MPTP group, thus suggesting a strong reduction in bradykinesia (Fig. 1A and B). A slight but significant reduction was also found in SUN11602 2.5 mg/kg group (Fig. 1A and B). Contrarily, SUN11602 1 mg/kg did not show considerable improvement in the behavioral test (Fig. 1A and B). EMP reported an increase in the percentage of time spent in the closed arm by MPTP-intoxicated mice compared to the Sham group (Fig. 1C and D). Instead, it was interesting to note that SUN11602 2.5 mg/kg, and especially at the dose of 5 mg/kg, effectively reduced the time spent in the closed arms compared to MPTP mice (Fig. 1C and D). There was no significant improvement with SUN11602 1 mg/kg administration (Fig. 1C and D). Sham + SUN11602 administered groups were comparable to Sham + vehicle animals (Fig. 1A–D).

### SUN11602 administration reduced loss of TH expression following MPTP injection

**Fig. 2:**
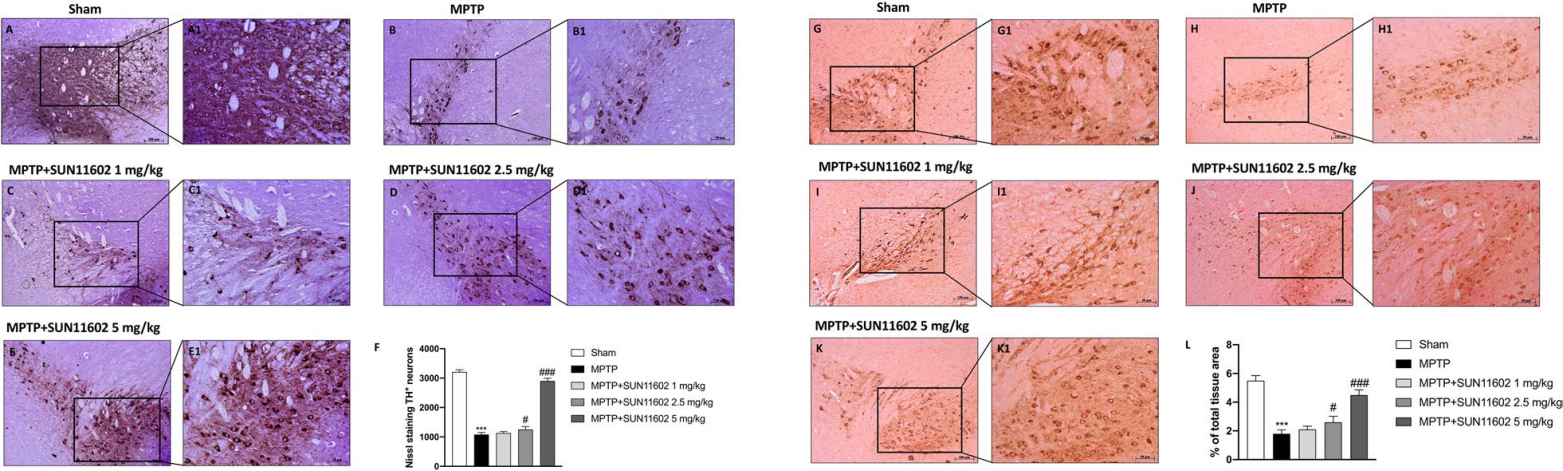
Effect of SUN11602 treatment on TH expression. MPTP mice exhibited an extensive loss of TH^+^ neurons (**B, B1, score F**), compared to the Sham group (**A, A1, score F**). SUN11602 2.5 mg/kg treatment, and especially at the dose of 5 mg/kg, restored the numbers of TH^+^ neurons (**D, D1; E, E1, score F**). SUN11602 1 mg/kg was ineffective (**C, C1, score F**). MPTP-injured mice revealed a marked loss of TH^+^ cells (**H, H1,** **score** **L**) compared to the Sham group (**G, G1, score L**). SUN11602 2.5 mg/kg, and more powerfully 5 mg/kg, counteracted the degeneration of dopaminergic neurons (**J, J1; K, K1, score L**). SUN11602 1 mg/kg proved to be ineffective (**I**, **I1, score L**). Data are representative of at least three independent experiments. Values are means ± SEM. One-way ANOVA test. ***p < 0.001 vs Sham; ^#^*p* < 0.05 vs MPTP; ^###^*p* < 0.001 vs MPTP

In order to confirm the neuroprotective effect of SUN11602 after MPTP intoxication, we evaluated TH expression by stereological analysis and immunohistochemical staining. As evidenced by stereological analysis MPTP-injected mice exhibited a substantial loss in the number of TH^+^ neurons (Fig. 2B and B1, score Fig. 2F) compared to the Sham animals (Fig. 2A and A1, score Fig. 2F). SUN11062 1 mg/kg administration did not induce any considerable increase (Fig. 2C and C1, score Fig. 2F). Contrarily, SUN11602 treatment at the dose of 2.5 mg/kg (Fig. 2D and D1, score Fig. 2F), and especially at 5 mg/kg, considerably preserved the number of TH^+^ neurons (Fig. 2E and E1, score Fig. 2F).

A decrease of TH-positive neurons was observed 7 days after MPTP injection (Fig. 2H and H1, score Fig. 2L) compared to the Sham group (Fig. 2G and G1, score Fig. 2L). SUN11602 1 mg/kg treatment did not show any significant improvement (Fig. 2I and I1, score Fig. 2L). However, SUN11602 treatment at the dose of 2.5 mg/kg, and in a more significant way at 5 mg/kg, restored TH expression levels (Fig. 2J, J1 and K, K1, respectively; score Fig. 2L).

### SUN11602 prevented dopamine transporter depletion from MPTP toxicity

**Fig. 3:**
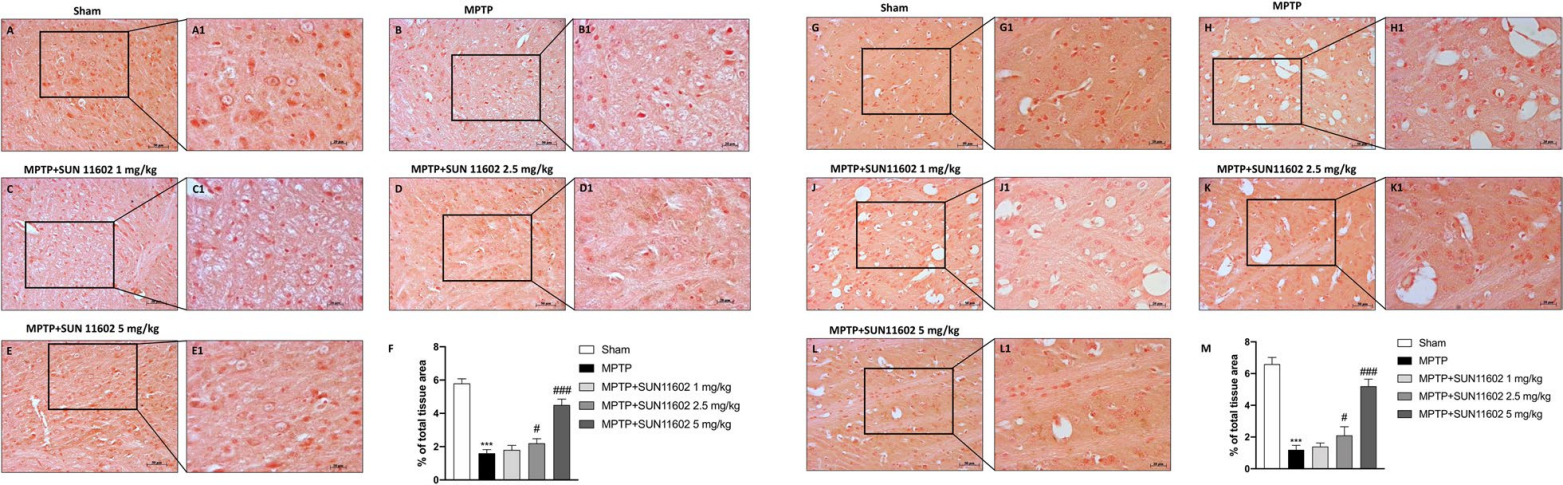
Effect of SUN11602 on DAT expression. MPTP-intoxicated mice showed a significant decrease of DAT expression in substantia nigra (**B, B1, score F**) compared to the Sham group (**A, A1, score F**). SUN11602 administration, at the doses of 2.5 (**D, D1, score F**) and more meaningfully at 5 mg/kg (**E, E1, score F**) resulted neuroprotective. SUN11602 1 mg/kg didn’t demonstrate significant efficacy (**C, C1, score F**). MPTP group showed a significant reduction of DAT expression in the striatum (**H**, **H1**, **score** **M**), compared to the Sham group (**G, G1, score M**). Dose-dependent response to DAT recovery was detected following treatment with SUN11602 (**J, J1, K, K1, L, L1, score M**). Data are representative of at least three independent experiments. Values are means ± SEM. One-way ANOVA test. ***p < 0.001 vs Sham; ^#^*p* < 0.05 vs MPTP; ^###^*p* < 0.001 vs MPTP

We evaluated DAT expression in the substantia nigra in order to investigate the neuroprotective effects of SUN11602 treatment on the dopamine pathway. A significant loss of DAT-positive staining was detected in MPTP-injected mice (Fig. 3B and B1, score Fig. 3F) compared to the Sham group (Fig. 3A and A1, score Fig. 3F). The restoration of DAT levels was remarkable following SUN11602 2.5 mg/kg administration (Fig. 3D and D1, score Fig. 3F) and more effective after SUN11602 5 mg/kg treatment (Fig. 3E and E1, score Fig. 3F), compared to the MPTP group. While, SUN11602 1 mg/kg treatment did not improve meaningfully DAT expression (Fig. 3C and C1, score Fig. 3F).

Moreover, considering the striatum a major locus of dopamine action, we also evaluated DAT expression in this brain area by immunohistochemical localization. MPTP-intoxicated mice exhibited a significant reduction in DAT expression (Fig. 3H, H1, score M), compared to the Sham group (Fig. 3G, G1, score Fig. 3M). SUN11602 2.5 mg/kg treatment, (Fig. 3K and K1, score Fig. 3M) and in a more significant way SUN11602 5 mg/kg administration (Fig. 3L and L1, score Fig. 3M), showed a considerable restoration of DAT levels. SUN11602 1 mg/kg did not induce significant increases in DAT levels (Fig. 3J, J1, score Fig. 3M).

### Effect of SUN11602 on dopamine metabolites after MPTP intoxication

**Fig. 4:**
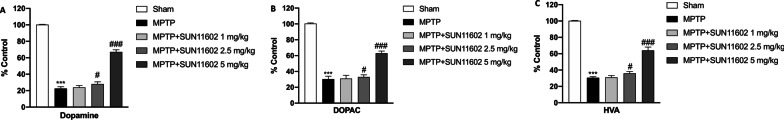
Effect of SUN11602 on dopamine metabolites after MPTP intoxication. MPTP-injected animals exhibited a considerable loss of dopamine and its metabolites, compared to the Sham mice; contrarily, treatment with SUN11602 in a dose-dependent manner increased metabolites levels (**A**–**C**). Data are representative of at least three independent experiments. Values are means ± SEM. One-way ANOVA test. ***p < 0.001 vs Sham; ^#^*p* < 0.05 vs MPTP; ^###^*p* < 0.001 vs MPTP

Degeneration of the nigrostriatal innervation leads to the loss of dopaminergic cells. Therefore, we evaluated the effect of SUN11602 on dopamine metabolism, measuring the striatal levels of dopamine and its metabolites: DOPAC and HVA. MPTP intoxication reduced striatal dopamine, DOPAC, and HVA levels which appeared around 20%, respectively. SUN11602 1 mg/kg treatment did not block this MPTP-induced loss. Differently, SUN11602 2.5 mg/kg, and especially at 5 mg/kg, showed significant restoration of dopamine and its metabolites levels, which were around 65% (Fig. 4A–C).

### Effect of SUN11602 on α-synuclein accumulation induced by MPTP intoxication

**Fig. 5:**
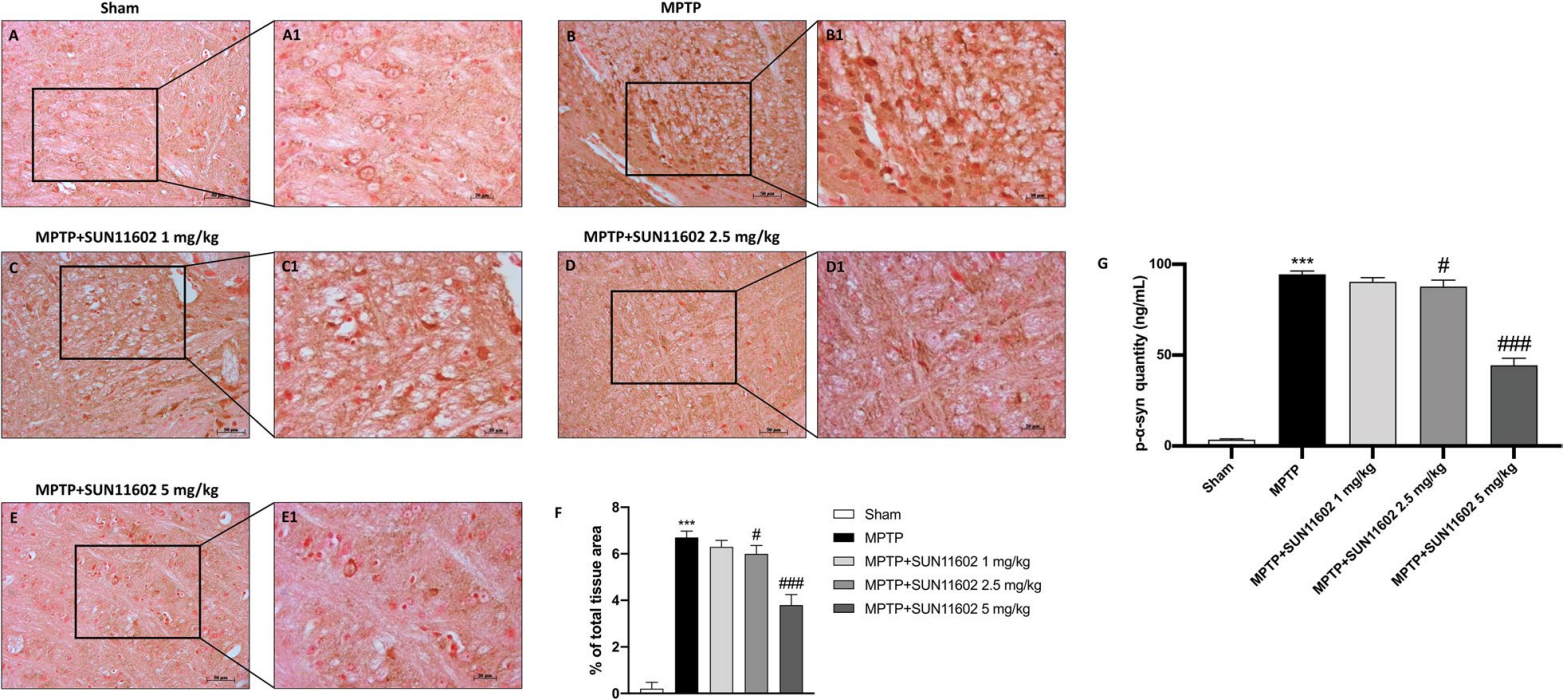
SUN11602 administration preserved α-Synuclein accumulation. MPTP-intoxicated showed an increase in the number of α-syn aggregates in dopaminergic neurons (**B, B1, score F**), compared to the Sham group (**A, A1, score F**). The neuroprotective role of SUN11602, at doses of 2.5 mg/kg and 5 mg/kg, in MPTP-injected mice is revealed by the reduction in the number of α-syn aggregates (**D, D1** and **E, E1, score F**). SUN11602 1 mg/kg was ineffective in diminishing α-Synuclein accumulation (**C, C1, score F**). Concordant results were also obtained from ELISA analysis of p-α-syn (**G**). Data are representative of at least three independent experiments. Values are means ± SEM. One-way ANOVA test. *** *p* < 0.001 vs Sham; # *p* < 0.05 vs MPTP; ### *p* < 0.001 vs MPTP

Aggregation of cytoplasmatic α-syn in the dopaminergic neurons of the substantia nigra is a typical PD characteristic [28]. To assess the neuroprotective effect of SUN11602, we performed immunohistochemistry staining. Here, we demonstrated that SUN11602, at the dose of 2.5 mg/kg (Fig. 5D, D1, score Fig. 5F), and more considerably at 5 mg/kg (Fig. 5E, E1, score Fig. 5F), was able to counteract the deposition of α-syn in dopaminergic neurons compared to MPTP mice (Fig. 5B, B1, score Fig. 5F), thus playing a neuroprotective role. SUN11602 1 mg/kg didn’t demonstrate significant efficacy (Fig. 5C, C1, score Fig. 5F). Sham group showed basal levels of α-syn (Fig. 5A, A1, score Fig. 5F).

To further assess the neuroprotective effect of SUN11602 against α-syn accumulation, we also evaluated p-α-syn form as peculiar protein implicated in the pathogenesis of PD. Elevated p-α-syn levels were found in the MPTP group compared to the Sham mice  (Fig. 5G). SUN11602, in a dose-dependent manner, decreased p-α-syn levels, resulting particularly effective at the dose of 5 mg/kg (Fig. 5G).

Considering these preliminary results, we decided to continue our experiments analyzing only SUN11602 5 mg/kg as the most effective dose in counteracting MPTP-induced nigrostriatal degeneration.

### Effect of SUN11602 treatment on GFAP, IBA-1and CD68 expression

The MPTP-induced nigrostriatal dopaminergic degeneration in neurons is accompanied by a substantial increase of astrocyte and microglia activation, which results in an upregulation of the respective markers GFAP and IBA-1 as well as CD68.

**Fig. 6:**
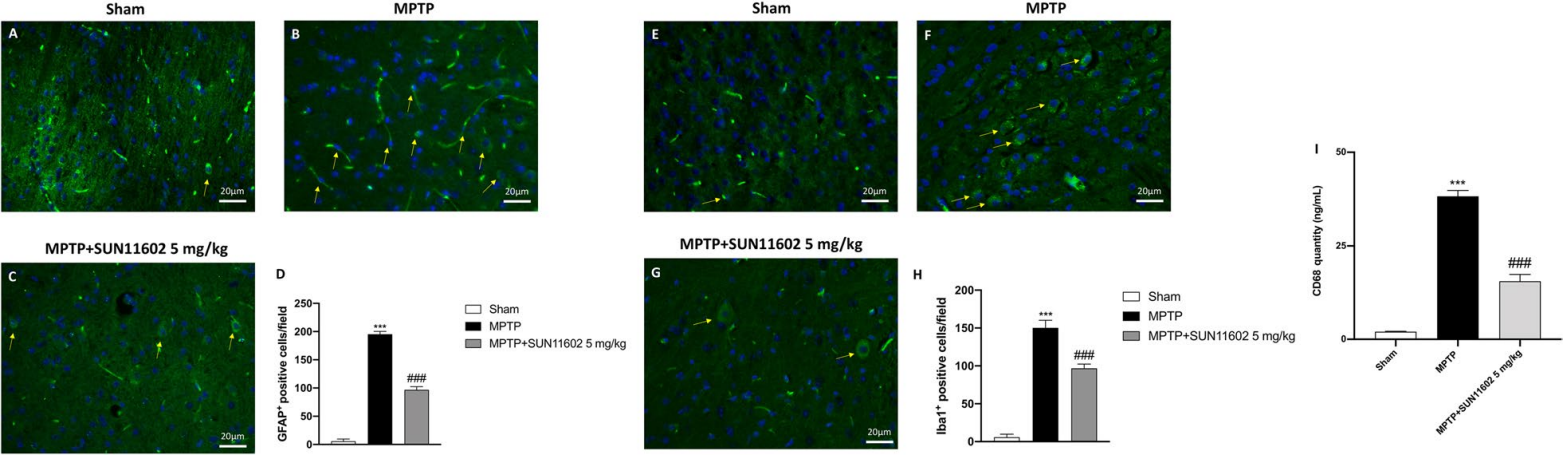
Effect of SUN11602 on GFAP, IBA-1 and CD68 expression. Tissues of MPTP-injected mice showed high expression of GFAP (**B, score D**) and IBA-1 (**F, score H**), compared to the Sham groups (**A**, **score** **D**; **E**, **score** **H**). GFAP and IBA-1 expression decreased after SUN11602 5 mg/kg administration (**C**, **score** **D**, and **G**, **score** **H**, respectively). SUN11602 also decreased CD68 levels (**I**). Data are representative of at least three independent experiments. Values are means ± SEM. One-way ANOVA test. ***p < 0.001 vs Sham; ^###^*p* < 0.001 vs MPTP

Immunofluorescence staining revealed that GFAP and IBA-1 were significantly higher in the MPTP group (Fig. 6B and F, respectively, score Fig. 6D and H) compared to the control group (Fig. 6A and E, respectively, score Fig. 6D and H) thus denoting reactive astrocyte and microglia. We investigated whether the neuroprotective effects of SUN11602 were associated with the attenuation of these pro-inflammatory markers. Our data reported that the number of GFAP and IBA-1 positive cells was significantly reduced in SUN11602-treated mice (Fig. 6C and G, respectively, score Fig. 6D and H). Furthermore, we evaluated CD68 levels as additional marker of reactive microglia through ELISA kit. The results confirmed the ability of SUN11602 to decrease microglia reactivity (Fig. 6I).

### SUN11602 modulated NF-kB pathway and reduced pro-inflammatory cytokines levels induced by MPTP intoxication

**Fig. 7:**
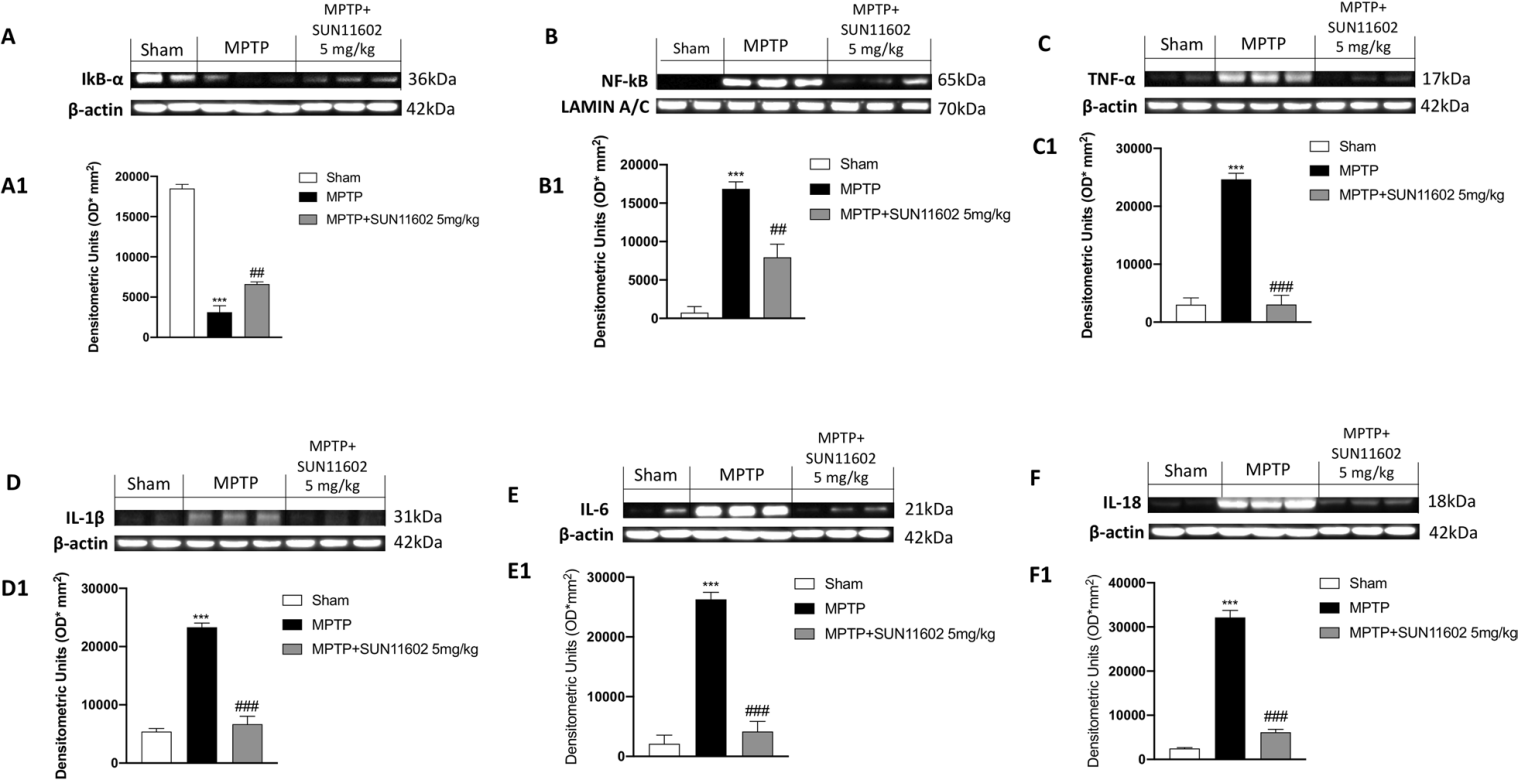
SUN11602 administration reduced neuroinflammation after MPTP injection. Western Blot analysis revealed a decreased expression of IĸB-α in MPTP-injected mice, compared to the Sham group (**A, densitometric analysis A1**). NF-kB levels resulted elevated in the MPTP-intoxicated mice compared to Sham animals (**B, densitometric analysis B1**). The anti-inflammatory effects of SUN11602 5 mg/kg were confirmed by increased IKB-α levels and at the same time by reduced NF-kB expression (**A, A1; B, B1**). A significant upregulation of pro-inflammatory cytokines expression was detected in the MPTP group, compared to the Sham mice (**C**–**F**, **densitometric analysis** **C1**–**F1**). These expressions were considerably reduced following SUN11602 5 mg/kg treatment (**C**–**F**, **densitometric analysis** **C1**–**F1**). Data are representative of at least three independent experiments. Values are means ± SEM. One-way ANOVA test. ***p < 0.001 vs Sham; _##_*p* < 0.01 vs MPTP; ^###^*p* < 0.001 vs MPTP

In PD, neuroinflammation plays an important role in the neurodegenerative process and the consequent loss of dopaminergic neurons. For this purpose, we evaluated the expression of IκB-α and the nuclear translocation of NF-κB p65 by Western Blot analysis. In the MPTP group we detected an extensive degradation of IĸB-α levels, compared to the Sham group, in which basal expression of IĸB-α was found (Fig. 7A, see densitometric analysis A1). However, SUN11602 5 mg/kg administration significantly restored IκB-α levels (Fig. 7A, see densitometric analysis A1).

On the other hand, we observed an increase in the nuclear translocation of NF-κB p65 in MPTP-intoxicated mice compared to the Sham group (Fig. 7B, see densitometric analysis B1). Such increase was considerably reduced by SUN11602 5 mg/kg treatment (Fig. 7B, see densitometric analysis B1).

NF-κB is considered crucial for initiating the inflammatory signaling pathway, since it leads to the activation of several pro-inflammatory factors such as IL-1β, IL-6, IL-18 and TNF-α [10].

Our data showed a significant upregulation of IL-1β, IL-6 and IL-18 and TNF-α in MPTP-intoxicated mice compared to the Sham group (Fig. 7C–F; see densitometric analysis Fig 7C1-F1). SUN11602 5 mg/kg administration demonstrated its ability to reduce cytokines levels (Fig. 7C–F; see densitometric analysis Fig 7C1-F1).

### Effect of SUN11602 treatment on microtubule organization and intracellular calcium homeostasis after MPTP intoxication

Calcium is involved in maintaining the basal activity of the Central Nervous System (CNS), the alteration of its cytoplasmic concentration triggers biochemical events that cause cell damage [29]. Calbindin-D28k and S-100β are important calcium-binding proteins involved in the homeostasis of Ca^2+^ and the regulation of cellular processes, such as cell progression and differentiation.

**Fig. 8:**
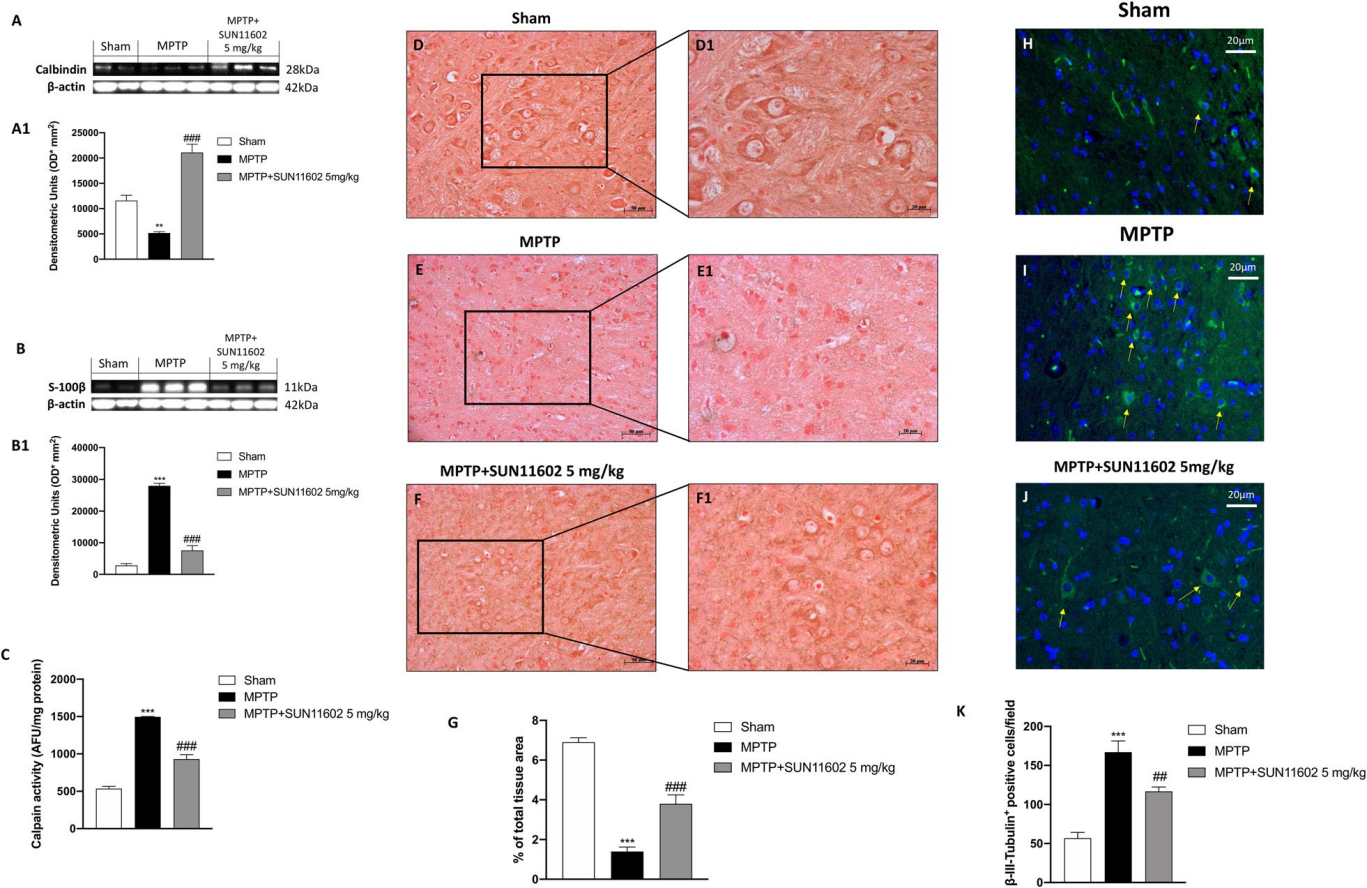
Effect of SUN11602 on neurons architecture and calcium homeostasis following MPTP injection. MPTP-injected mice showed an important decrease of Calbindin expression compared to the Sham group; while SUN11602 induced a significant upregulation of Calbindin (**A, densitometric analysis A1**). S-100β and Calpain levels were increased in MPTP-intoxicated mice, however, SUN11602 administration was able to reduce their levels (**B, densitometric analysis B1, C**). MPTP-intoxicated mice exposed low expression of MAP-2 (**E, E1, score G**) compared to the Sham group (**D, D1, score G**). SUN11602 5 mg/kg was able to restore the levels of MAP-2 protein (**F, F1, score G**). MPTP-injured mice exposed an increase of β3-tubulin-positive cells (**I, score K**) compared to the Sham animals (**H, score K**), SUN11602 5 mg/kg administration decreased the non-physiological β3-tubulin expression (**J, score K**). Data are representative of at least three independent experiments. Values are means ± SEM. One-way ANOVA test. ***p < 0.001 vs Sham; ^##^*p* < 0.01 vs MPTP; ^###^*p* < 0.001 vs MPTP

Elevated levels of S-100β and Calpain were reported in neurodegenerative diseases. Accordingly, our results showed an important decrease in Calbindin-D28k expression in MPTP-injected mice (Fig. 8A, see densitometric analysis Fig. 8A1). Conversely, SUN11602 5 mg/kg treatment increased, in a significant way, Calbindin-D28k levels (Fig. 8A, see densitometric analysis Fig. 8A1). S-100β and Calpain, markers of brain damage, were increased in the MPTP group, compared to the Sham animals (Fig. 8B, see densitometric analysis Fig. 8B1 and C, respectively). SUN11602 5 mg/kg treatment was able to significantly reduce S-100β and Calpain levels (Fig. 8B, see densitometric analysis Fig. 8B1 and C, respectively), demonstrating a good capability to improve brain damage (Additional file 1).

The involvement of two important neuronal markers like MAP-2 and β3-tubulin was investigated by immunohistochemical and immunofluorescence staining, respectively. MAP-2 is a cytoskeletal protein, its role is to stabilize the assembly of microtubules, therefore it is considered a marker of synaptic plasticity [30]. Brain tissues from MPTP-injected mice exhibited a reduction of MAP-2 levels (Fig. 8E and E1, score Fig. 8G), compared to the Sham group (Fig. 8D and D1, score Fig. 8G). SUN11602 5 mg/kg treatment restored the MAP-2 expression (Fig. 8F and F1, score Fig. 8G), counteracting the progression of neuronal loss.

Moreover, we also examined β3-tubulin expression, as a constitutive protein of the neuronal cell cytoskeleton [8]. We observed an extensive increase in the number of β3-tubulin-positive cells in the MPTP group (Fig. 8I, score Fig. 8K), compared to the Sham group (Fig. 8H, score Fig. 8K). SUN11602 5 mg/kg administration demonstrated its good capacity to decrease the number of β3-tubulin-positive cells (Fig. 8J, score Fig. 8K).

### Effect of SUN11602 treatment on apoptosis pathway following MPTP injection

Neuroinflammation and Ca^2+^ excitotoxic damage are predisposing factors to cell death, which impact neurodegenerative diseases through apoptosis or necrosis processes [31].

In this context, we evaluated anti/pro-apoptotic markers like Bcl-2, Bax and Caspase-3 by Western Blot analysis, and p53 by immunofluorescence analysis.

**Fig. 9:**
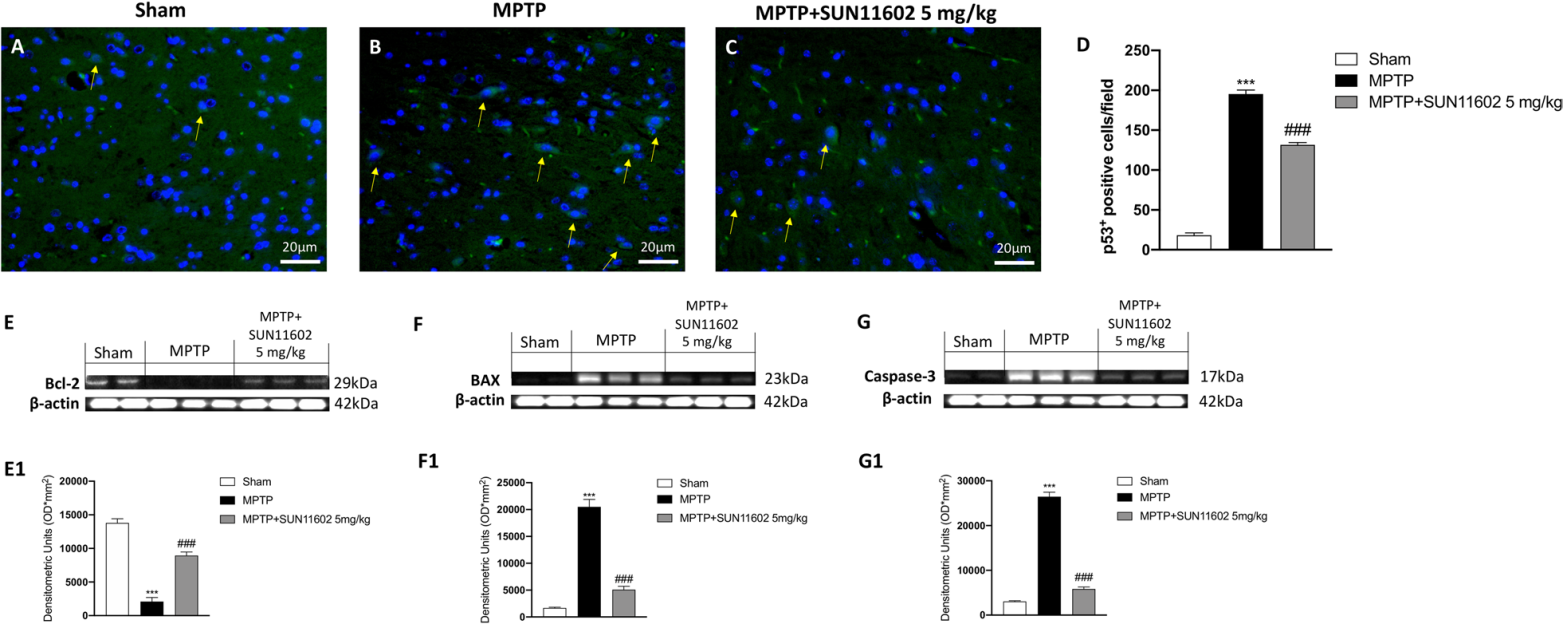
SUN11602 administration modulated apoptosis after MPTP injection. Midbrain section obtained by MPTP-injected mice exposed high expression of p53 (**B**, **score** **D**), compared to the Sham animals (**A**, **score** **D**). SUN11602 5 mg/kg treatment demonstrated a significant reduction in p53 levels (**C**, **score** **D**). MPTP-intoxicated mice displayed increased expression of Bax and Caspase-3 and diminished levels of Bcl-2, compared to the Sham group (**E**, **densitometric analysis** **E1**; **F, densitometric analysis F1; G, densitometric analysis G1**). SUN11602 5 mg/kg administration reduced the expression of Bax and Caspase-3 while increasing Bcl-2 levels (**E, densitometric analysis E1; F, densitometric analysis F1; G, densitometric analysis G1**). Data are representative of at least three independent experiments. Values are means ± SEM. One-way ANOVA test. ***p < 0.001 vs Sham; ###*p* < 0.001 vs MPTP

The p53 protein plays a physiological role in cell cycle control. Indeed, following damage or an injury p53 controls the apoptotic cascade, causing neuronal death. Immunofluorescence staining showed an increase of p53 expression in the MPTP-intoxicated mice (Fig. 9B, score Fig. 9D), compared to the Sham group in which p53 is physiologically expressed (Fig. 9A, score Fig. 9D). SUN11602 5 mg/kg treatment demonstrated a significant reduction in p53 levels, thus suggesting its neuroprotective role against the apoptotic cascade (Fig. 9C, score Fig. 9D).

Bcl-2 levels are reduced after MPTP injection (Fig. 9E, see densitometric analysis Fig. 9E1). Instead, the expressions of Bax (Fig. 9F, see densitometric analysis Fig. 9F1) and Caspase-3 (Fig. 9G, see densitometric analysis Fig. 9G1) were increased in the MPTP-intoxicated mice compared to the Sham group. SUN11602 5 mg/kg administration was effective in reducing Bax and Caspase expressions while restoring Bcl-2 levels (Fig. 9E, see densitometric analysis Fig. 9E1; Fig. 9F, see densitometric analysis Fig. 9F1; Fig. 9G, see densitometric analysis Fig. 9G1). These data suggest the neuroprotective role of SUN11602 in counteracting the apoptotic processes.

## Discussion

PD is a progressive neurodegenerative disease whose pathological characteristic is the degeneration of the nigrostriatal dopaminergic system [32]. Current therapeutic approaches of Parkinsonian patients aimed to attenuate clinical symptoms through the administration of levodopa and monoamine oxidase B (MAO-B) inhibitors [33, 34]. However, although conventional treatments proved to be valuable in symptoms relief, they do not represent a resolutive cure for PD [35]. Thus, the care of PD patients represents a worldwide problem whereby is required to develop more effective therapies. In this perspective, the deepening of PD pathological background may result helpful in the discovery of new therapeutic targets as well as novel therapies for the management of PD patients. Recently, the role of neuroinflammation in PD has aroused great interest from the scientific world. Indeed it was emphasized the role of glial activation, cytokine production, impaired calcium homeostasis, and apoptosis signaling in the progressive degeneration of dopaminergic neurons [18, 36]. Hence, targeting the signaling pathways responsible for neuroinflammation may represent a promising approach to improve neurons survival in PD [37]. In particular, bFGF/FGFR1 axis was reported to play an important neuroprotective role in CNS disorders [38]. Unfortunately, a clinical study using native bFGF in acute stroke patients failed to demonstrate safety, given the observation of several side effects [16]. Thus, it has been synthetized bFGF functional analogues to develop safer drugs [13].

The novel synthetic compound SUN11602 activates various signaling factors, leading to neuroprotective effects similarly to bFGF [13, 16].

Therefore, on these bases, the purpose of the present study was to evaluate the neuroprotective abilities of SUN11602, a bFGF mimetic, in an MPTP-mouse model of nigrostriatal degeneration.

Motor and non-motor dysfunctions are clinicopathological signs of PD patients. It has been well known that PD is characterized by a variety of behavioral deficits, resulting in an extensive symptomatology concerning motor control, such as bradykinesia, rigidity, and tremor [39].

In addition to this, cognitive impairments and emotional deficits consistently affect the well-being of PD patients, thus worsening their clinical picture. Our data clearly indicated a decline of motor and non-motor functions in MPTP-injected mice. Otherwise, SUN11602 treatment effectively provided symptom relief by promoting the recovery of motor and non-motor behavioral functions, preventing the loss of locomotor agility, and improving mice’s emotional state.

Behavioral disturbances are a manifestation of an impaired dopaminergic system [40]. In these complex circuits, TH plays a key role. This enzyme catalyzes the conversion of l-tyrosine to l-3,4-dihydroxyphenylalanine (L-DOPA), which represents the initial and rate-limiting step in the biosynthesis of catecholamines [41]. Moreover, as revealed by several post-mortem studies, the alteration in TH content has a predictive validity to estimate the risk of PD progression as well as catecholamine dysfunction [42]. Accordingly, our results showed a marked decrease in TH expression in MPTP-injured mice. Differently, oral administration of SUN11602 considerably prevented TH^+^ expression in neurons, protecting against MPTP-induced damage in the CNS.

The survival of nigral neurons is closely linked to the preservation of nigrostriatal dopamine homeostasis; in particular, this process is interconnected to the nerve cells’ capability to synthesize, store and release dopamine [43]. In this context, the regulation of dopamine feedback signals by DAT provides the neurons the ability to modulate dopamine clearance in response to physiological demands [44]. More specifically, DAT function results in rapid take up of dopamine from the extracellular space into the presynaptic neuron, essential for regulating the magnitude and duration of dopaminergic signaling [45]. The results obtained from this study highlighted a substantial decrease in DAT expression both in the substantia nigra and in the striatum after MPTP administration. Instead, SUN11602-treated mice revealed a considerable upregulation of DAT levels in brain areas, thus supporting the neuroprotective capabilities of this bFGF mimetic.

Moreover, to further verify the effect of SUN11602 on dopaminergic neuron loss we assessed the levels of dopamine and its metabolites such as DOPAC and HVA in the striatum. We observed that SUN11602 administration counteracted the dramatic reduction of striatal dopamine, DOPAC and HVA levels after MPTP injection, thus denoting positive outcomes on dopaminergic neural networks.

Among the hallmarks of PD, α-synuclein acts as a central component, causing synaptic toxicity and contributing to neuronal death.

Certainly, the accumulation of α-syn aggregates in neuronal cell bodies largely contributes to the degeneration of dopaminergic neurons, thus worsening cognitive decline in PD patients [18, 46]. From our study, an increased expression of pan-α-syn was observed in MPTP-injured mice. Nevertheless, SUN11602 treatment was able to reduce synucleinopathy induced by MPTP intoxication. The predominant species of the toxic aggregates is α-syn abnormally phosphorylated, a pathological modification that facilitates fibril formation and insoluble aggregation [47].

Thereby, considering the phosphorylation of α-syn critical in PD pathogenesis, we assessed its expression after SUN11602 treatment. Our data clearly highlighted a significant decrease in p-α-syn in SUN11602-treated mice, validating once again the beneficial effects of this compound.

In the CNS, glial cells, such as microglia and astrocytes, are generally correlated with supporting functions including energy metabolism, synaptic plasticity, and ion homeostasis [48].

However, in pathological conditions, the establishment of an unremitting neuroinflammatory state triggered the activation of glia, sustaining the mechanisms of neuronal degeneration in the onset of PD. Particularly, the increase in reactive astrocytes and microglia turned into elevated GFAP and IBA-1 expressions. Therefore, GFAP and IBA-1 represent reliable biomarkers of brain damage also in the context of neurodegenerative disorders.

The results obtained from this study clearly confirmed an extensive change in GFAP and IBA-1 expression after MPTP-induced nigrostriatal degeneration. Differently, SUN11602 administration attenuated reactive astrocytes and microglia, regulating GFAP and IBA-1 levels.

CD68 is also considered a marker of microglia, widely used to identify microglia activation stages in neuropathological analysis [49]. SUN11602 treatment was able to decrease CD68 levels, confirming its ability to decrease microglial activation.

In the multifaceted etiopathogenesis of PD, the inflammatory cascade is an important pathophysiological feature. Inflammation leads to disease progression through the activation of several pathways [1]. Concerning this, the transcriptional factor NF-κB is a key feature in orchestrating the manifold cellular activities underlying inflammation. This nuclear factor stimulates the release of numerous pro-inflammatory mediators such as enzymes, cytokines, and chemokines, which drive the exacerbation of the disease [50]. Thereby, the development of effective anti-inflammatory therapies, aimed at modulating NF-κB activity and its cross-talks, could constitute an interesting therapeutic approach in the protection of nigral neurons [50]. In the present study, SUN11602 administration exerted beneficial properties, effectively modulating the NF-κB pathway as well as cytokines expression. These data demonstrated the usefulness of SUN11602 in mitigating the neuroinflammatory condition.

Many papers have elucidated the bidirectional interactions between neuroinflammatory signaling mechanisms and Ca^2+^ dyshomeostasis [51]. Indeed, an increase of pro-inflammatory cytokines, such as TNF-α, IL-1β spreads the activity of voltage-sensitive L-type Ca^2+^ channels in neurons, impairing the regulation of intracellular Ca^2+^ [52]. In according, clinical studies indicated a compromise of cellular Ca^2+^-regulating systems in the field of neurodegenerative disorders [53]. Furthermore, these clinical data also suggest that calcium levels are responsible for the selective vulnerability of nigrostriatal dopaminergic neurons in PD [53].

In this circumstance, the modulation of Ca^2+^-binding proteins would emerge as a hopeful pharmacological strategy in controlling the shape and buffer of Ca^2+^ signals, supporting an excellent neuroprotective action [54]. Especially, Calbindin-D-(28 k) overexpression would protect dopaminergic neurons against the PD pathological process, inhibiting calcium-mediated calpain activation, and rebalancing Ca^2+^ overload in neurons [55, 56]. Consequently, considering the potential activity of SUN11602 in increasing Calbindin-D-(28 k) levels in neurons, we investigated its capability to restore calcium intracellular homeostasis after MPTP intoxication.

From the data obtained, we have proven that SUN11602 administration enhanced Calbindin-D-(28 k) expression, decreased S100-β levels and restrained Calpain activity. Thus, SUN11602 administration indicated a good handle of intracellular Ca^2+^ efflux through the regulation of Ca^2+^-binding proteins. Furthermore, literature data elucidated the pivotal function of Ca^2+^ in the regulation of neuronal synaptic plasticity as well as in controlling cytoskeleton formation [57, 58].

De facto, it has been well investigated how, in aging and disease, an imbalance in intracellular Ca^2+^ regulation leads to the degeneration of neural circuits through adaptive modification of the neural network. In light of this, it is clear how neuroarchitecture is influenced by the CNS environmental signals which regulate neural development. Calcium neuronal circuits alteration has a great impact on the different cytoskeletal systems.

Cytoskeletal associated proteins such as the MAP-2 protein represent a likely candidate in calcium regulation. MAP-2 would be able to limit the excessive excitotoxic activity and avoid aberrant neuroarchitectonic changes typical of neurodegenerative disorders.

On the other hand, scientific findings advised mutations in the structure of tubulin, the major microtubule constituent [59]. Particularly, it was highlighted an increase in the neuron-β3-tubulin isotype after MPTP intoxication [59]. Microtubules are essential for intracellular transport, thus their depolymerization may cause significant cell damage, especially for long projection neurons like dopamine cells of the nigrostriatal pathway [60].

In our study, SUN11602 administration restored the MPTP-induced MAP-2 loss while reducing β3-tubulin raises, in the number of labeled cells.

Ca^2+^ homeostasis disruption, due to an increase of free cytoplasmic Ca^2+^, can also induce cell death by activating apoptosis processes [61].

In this field, the tumor suppressor p53 is the main modulator of cellular stress responses. Following a wide range of insults, including cellular calcium overload and excitotoxicity, p53 production is rapidly increased in neurons.

Thus, in the CNS environment, p53 activation can trigger apoptosis in neurons by encoding the pro-apoptotic proteins Bax and Caspase-3. Based on these observations, we speculated that SUN11602 administration, modulating Ca^2+^ signaling, could prevent the apoptotic process, thus protecting neurons from neurodegeneration. Our results revealed that SUN11602 reduced p53, Bax and Caspase-3 levels, while Bcl-2 expression was significantly increased, thus confirming apoptosis modulation after MPTP-induced neuronal death.

## Conclusion

In conclusion, the data obtained from the present study emphasized the benefits provided by the SUN11602 administration. Our results demonstrated, for the first time, the neuroprotective effect of SUN11602 in a mouse model of MPTP-induced nigrostriatal degeneration.

Taken together, these positive outcomes translate into a better management of PD features, reduced neuroinflammation, improved Ca^2+^ disruption as well as neuronal cell death following MPTP-induced nigrostriatal degeneration. Considering these new insights, SUN11602 may represent a potential therapeutic approach in preserving the survival of dopaminergic neurons, thus constituting a valuable support in the pharmacological strategy for PD patients.

However, we are aware of the current PD animal models limitations, and in particular of the MPTP neurotoxin, in providing a useful platform for selectively studying the pathophysiology of PD. In fact, unlike clinical Parkinson's, the administration of MPTP produces only a rapid neurodegeneration, not completely recapitulating the PD symptomatology. Then, recognizing PD as a multifactorial disease, this study has some limitations related to the animal model used that may differ in another model.

Furthermore, since SUN11602 was administered only one day after the MPTP neurotoxin, further analyzes are required to understand whether or not SUN11602 affects the metabolism/clearance of MPTP/MPP^+^, resulting in reduced toxin levels.

Certainly, future targeted studies are needed to deepen the activity of this compound and confirm these preliminary results through well-designed clinical trials.

## Supplementary Information

**Additional file 1.** Full acquistions of Western Blots images are provided.

## Data Availability

All data in this study are included in this published article and its additional information files.

## Abbreviations

PD: Parkinson’s disease
bFGF/FGFR1: Basic fibroblast growth factor/fibroblast growth factor receptor-1
CNS: Central nervous system
TH: Tyrosine hydroxylase
DAT: Dopamine transporter
α-syn: α-Synuclein
L-DOPA: L-3,4-Dihydroxyphenylalanine
