# Supplementary material for: SUN11602, a bFGF mimetic, modulated neuroinflammation, apoptosis and calcium-binding proteins in an in vivo model of MPTP-induced nigrostriatal degeneration
Source: J Neuroinflammation. 2022 May 7;19:107. doi: 10.1186/s12974-022-02457-3 (PMC9080217; doi:10.1186/s12974-022-02457-3)

IkB-α

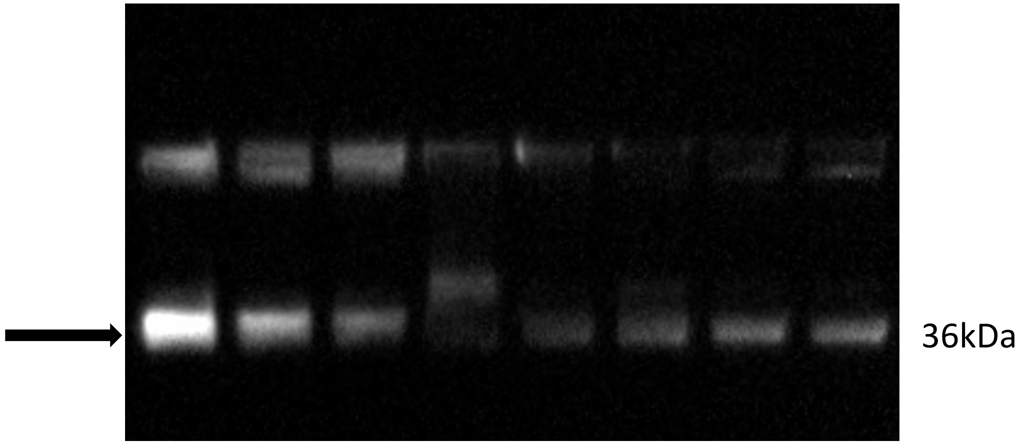

NF-kB

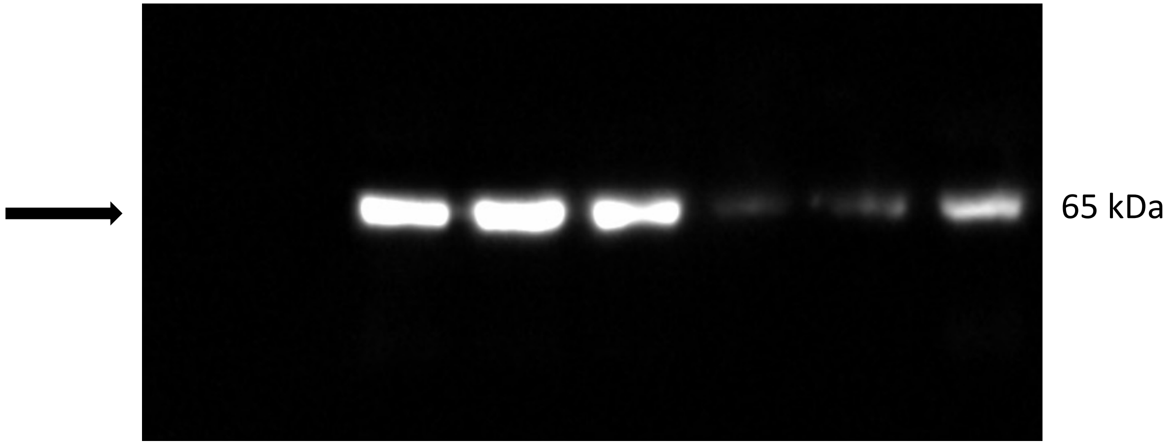

β-actin for IkB-α

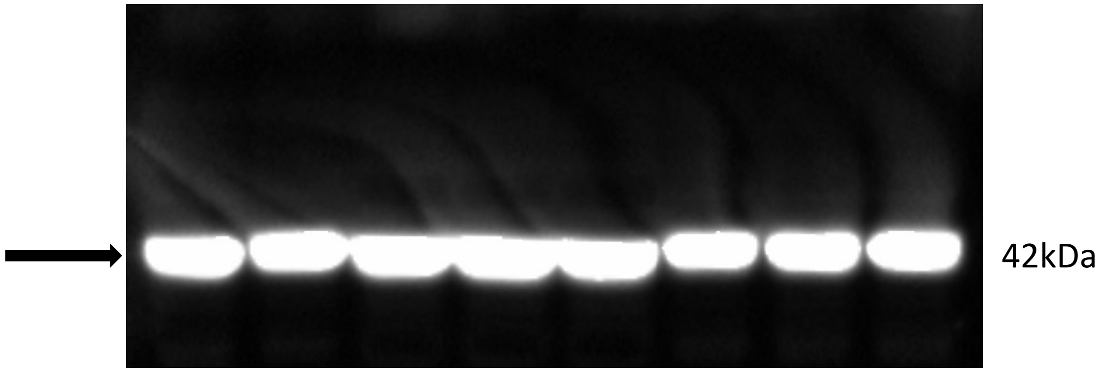

LAMIN A/C for NF-kB

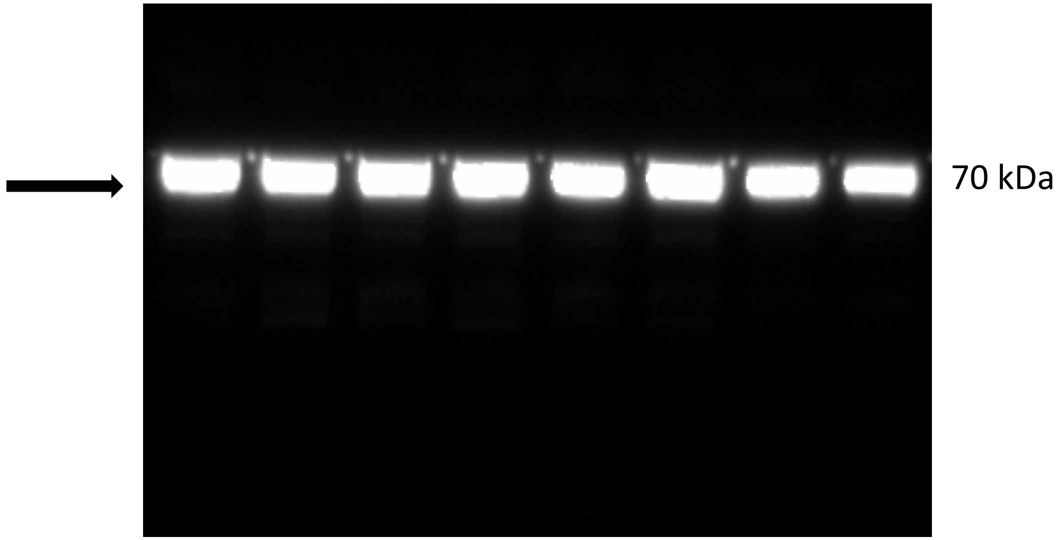

TNF-α

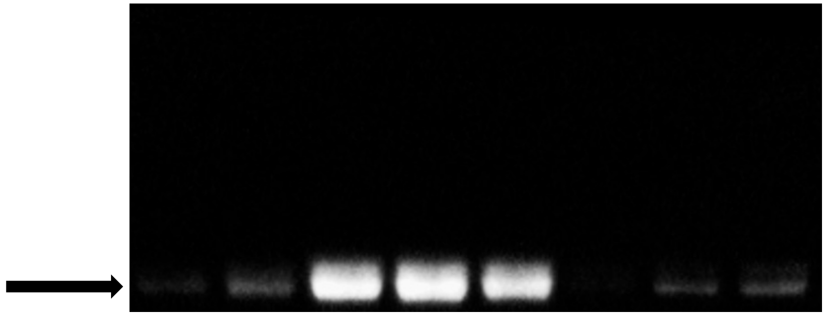

17kDa

IL1β

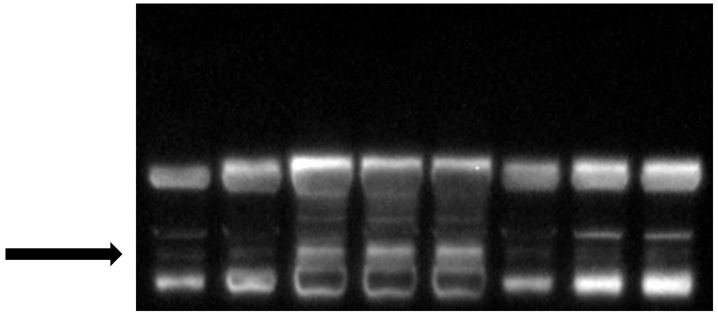

31kDa

β-actin

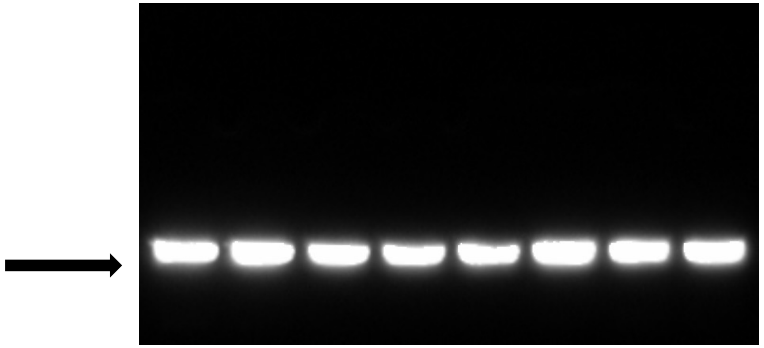

42kDa

IL-6

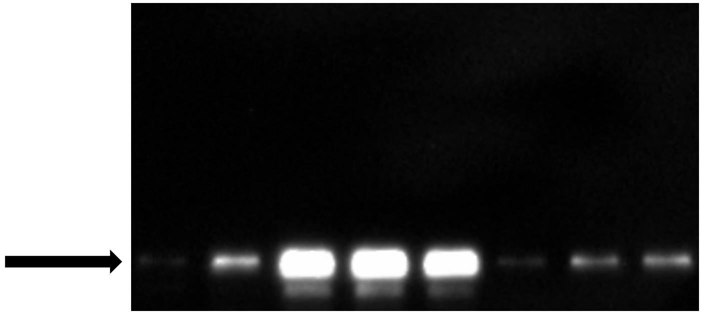

21kDa

IL-18

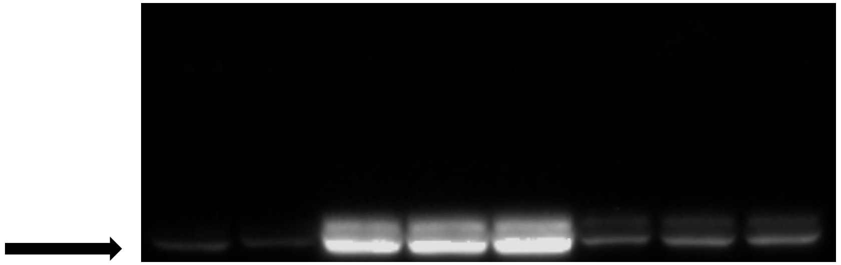

18kDa

β-actin for IL-6 and IL-18

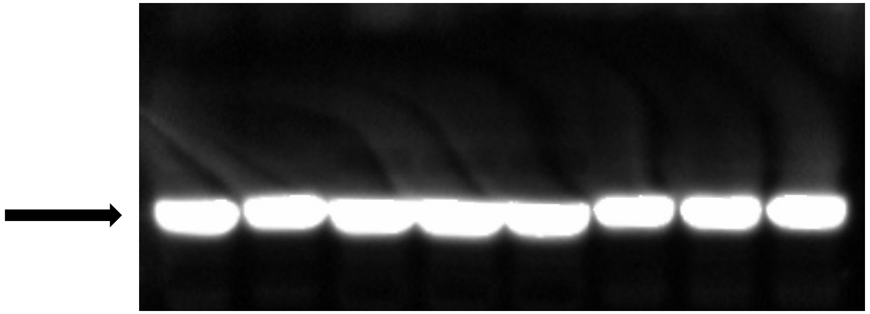

42kDa

Calbindin

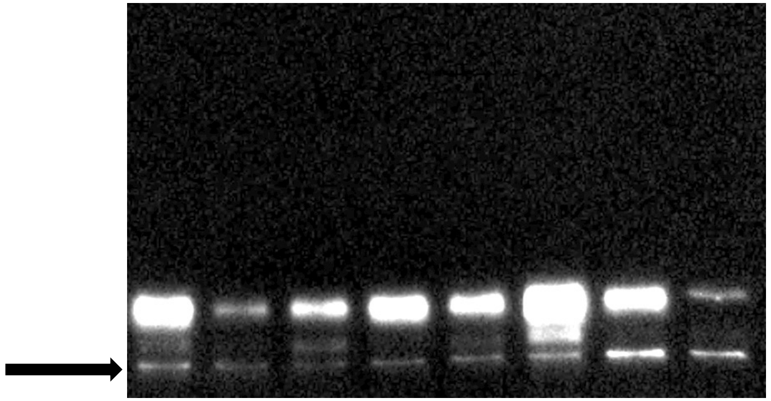

28kDa

S-100β

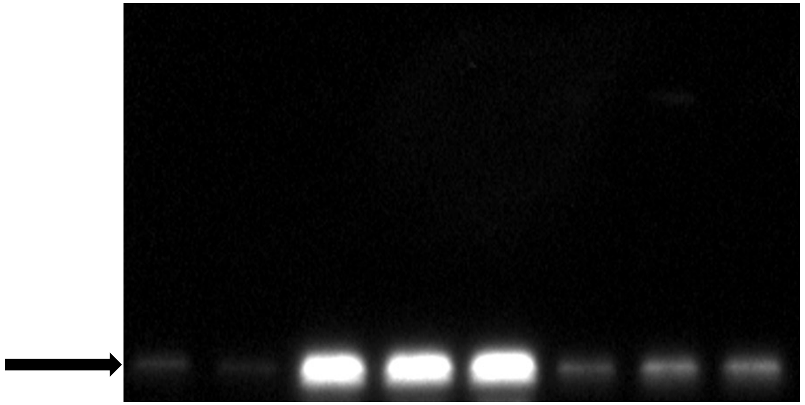

11kDa

β-actin for Calbindin and S-100β

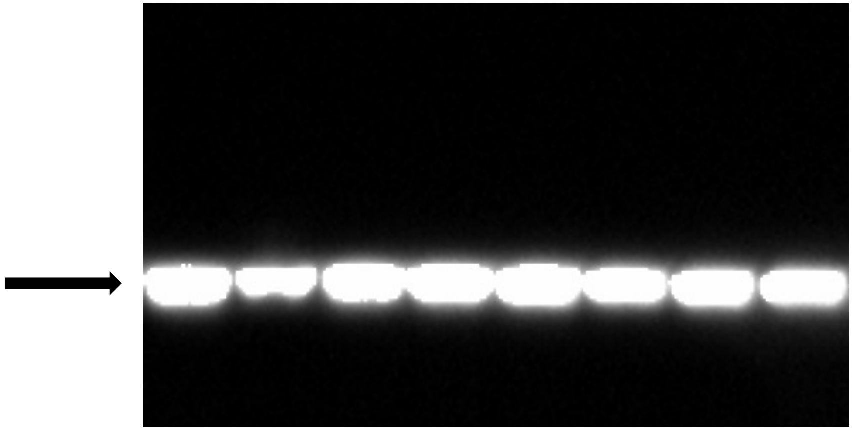

42kDa

BCL-2

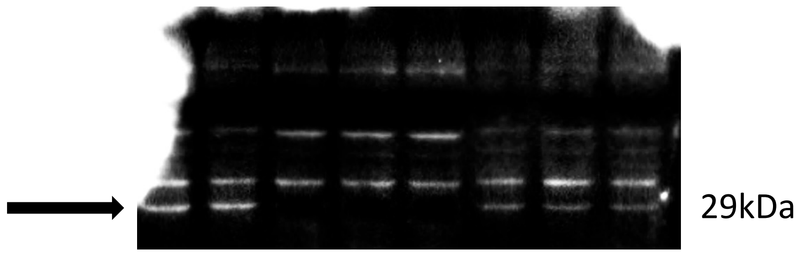

BAX

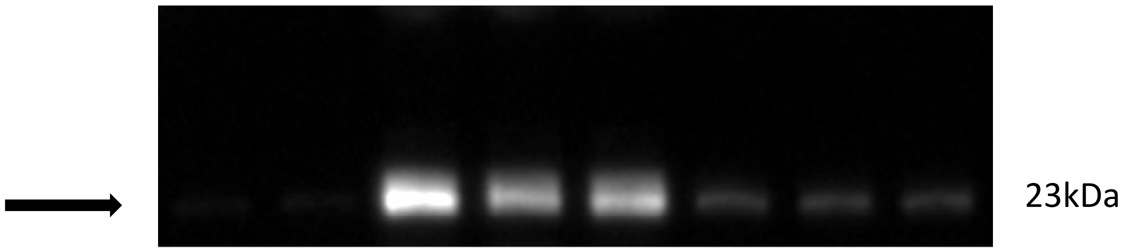

CASPASE-3

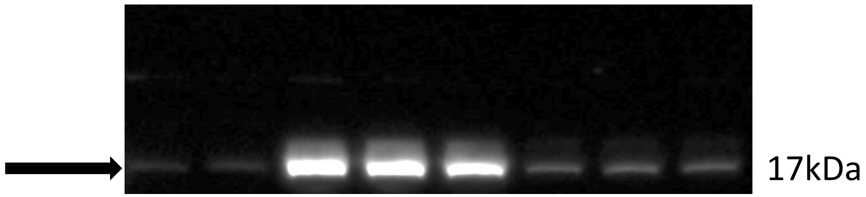

β-actin for BCL-2, BAX and CASPASE-3

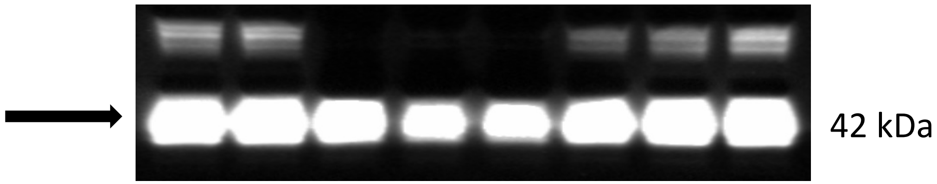

Supplement: Supplementary file 1 — Additional file 1. Full acquistions of Western Blots images are provided. [file 12974_2022_2457_MOESM1_ESM.pdf]
